# Isolated Pancreatic Tail Injury in Paediatrics; A Case Report and Literature Review 

**DOI:** 10.30476/beat.2020.85719

**Published:** 2020-10

**Authors:** Nabil Muhammad Al Kuddoos, Ahmad Khaldun Ismail, Kevin Wong Chuing Shen, Nur Amirah Shibraumalisi, Asmil Yuzairi Yunus

**Affiliations:** 1 *Department of Emergency Medicine, Faculty of Medicine, Hospital Canselor Tuanku Muhriz, Universiti Kebangsaan Malaysia Medical Centre, Jalan Yaacob Latif, Bandar Tun Razak, Cheras, Kuala Lumpur, Malaysia.*; 2 *Primary Care Medicine Department, Faculty of Medicine, UiTM, Selayang Campus, 68100* ***, *** *Batu Caves, Selangor * *Darul Ehsan* *, Malaysia*; 3 *Department of Radiology, Selayang Hospital, 68100, Batu Caves, Selangor * *Darul Ehsan* *, Malaysia*

**Keywords:** Pancreatic injury, Pancreatic trauma, Paediatrics, Malaysia

## Abstract

Pancreatic injury in paediatrics is a rare condition and can be difficult to diagnose. The diagnostic challenge is due to its symptom of vague abdominal pain which usually results in late presentation. Elevated biochemical markers such as amylase and lipase can aid in the evaluation of pancreatic injury, however, it is not specific and is only evident after several hours of trauma. Ultrasound is commonly used as a primary modality to evaluate abdominal organ injuries, but its role in detecting pancreatic injury is limited due to low sensitivity. High index of suspicion is needed to avoid undiagnosed pancreatic injury which could be lethal to children. We herein report a case of delayed presentation of isolated pancreatic tail injury in a child who was sufficiently diagnosed with ultrasound and treated conservatively. Proper initial assessment and diagnosis will allow appropriate management of pancreatic injury. Conservative management should include serial imaging to look at the evolution of pancreatic injury and detect complications such as pseudocyst or abscess formation.

## Introduction

Traumatic pancreatic injury in children accounts for 0.6% of all abdominal traumas with isolated pancreatic injuries occurring in less than one fifth of cases [1]. It mainly occurs in ages five to 18 years old and is the fourth most common abdominal organ injury in children [1, 2]. Majority of cases are associated with blunt trauma from motor vehicle accidents, direct blow from a fall or kick, and handlebar injuries [3]. Pancreatic trauma is difficult to be identified due to its retroperitoneal location especially in the presence of co-existing intra-abdominal organ injuries [4]. The initial symptom following isolated trauma to the abdomen is often vague or diffuse abdominal pain. Therefore, there should be a low threshold for the use of diagnostic imaging [1].

## Case Report

An 11-year-old girl presented to the emergency department (ED) with severe epigastric pain 10 hours following a direct blow to the abdomen by a seesaw handle at the playground. Initially the pain was tolerable but subsequently became unbearable associated with vomiting more than ten times. On arrival to ED, the pain was described as persistent and non-radiating. She had no significant past medical or surgical history. On examination, she was alert and orientated. Her blood pressure was 112/84 mmHg, heart rate was 112 beats per minute (bpm), respiratory rate was 20 breaths per minute, temperature was 37℃, and oxygen saturation of 98%. Her pain score was 10/10 in the epigastric region, with localised tenderness and guarding with normal bowel sounds. There was no bruising seen on the abdomen. Other systemic examinations were unremarkable. The initial impression was blunt abdominal trauma with hollow viscus injury. Focused assessment with sonography in trauma (FAST) revealed no free fluid in the peritoneal cavity and chest radiograph was normal. The pain was controlled with intravenous (IV) morphine 2 mg in titrated doses and IV tramadol 25 mg. Venous blood gas showed no acidosis with lactate level of 2.1 mmol/L. Her haemoglobin level was normal at 13.5 g/dL, white cell count was elevated at 15.3 x 10^9^/L and platelet count was elevated at 487 x 10^9/L. Liver function test (LFT) showed normal albumin level of 41 g/l, elevated total protein at 87 g/l, normal bilirubin of 8.6 µmol/l, normal alanine aminotransferase (ALT) of 25 U/L and elevated alkaline phosphatase (ALP) at 212 U/L. Her amylase level was significantly elevated at 2463 U/L. Her renal profile was normal.

**Fig. 1:**
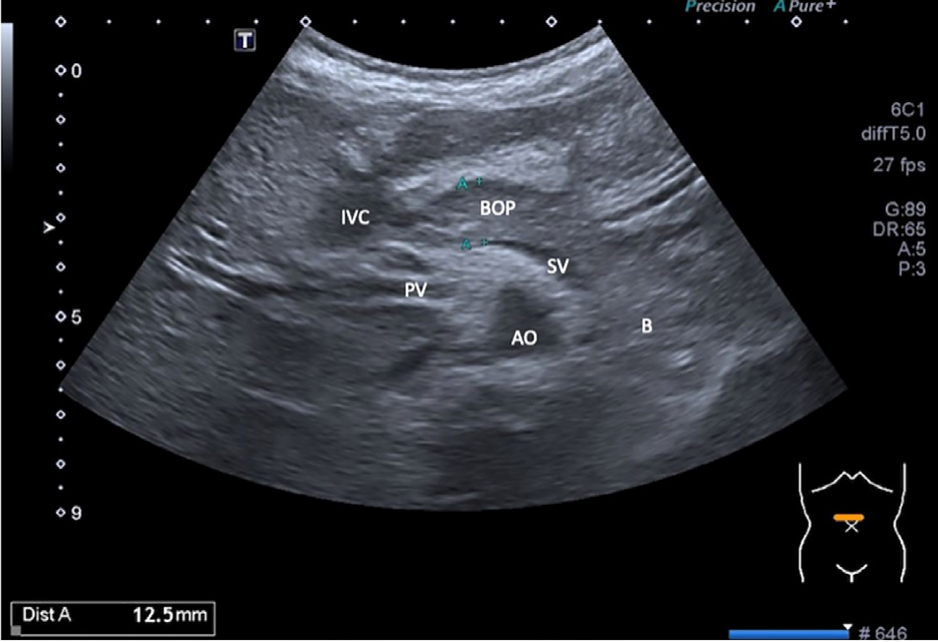
Transabdominal ultrasound mid axial plane at supraumbilical region showing homogenous mild hyperechoic appearance of pancreas which is normal for age and no evidence of laceration in the visualised neck and body of pancreas. The tail of pancreas is not visualised due to bowel gas obscuration. No peripancreatic free fluid. The rest of abdominal organs are unremarkable (not shown). **AO:** abdominal aorta, **B:** bowel, **SV:** splenic vein, **PV:** portal vein, **IVC:** intrahepatic inferior vena cava, **BOP:** neck and body of pancreas

**Fig. 2:**
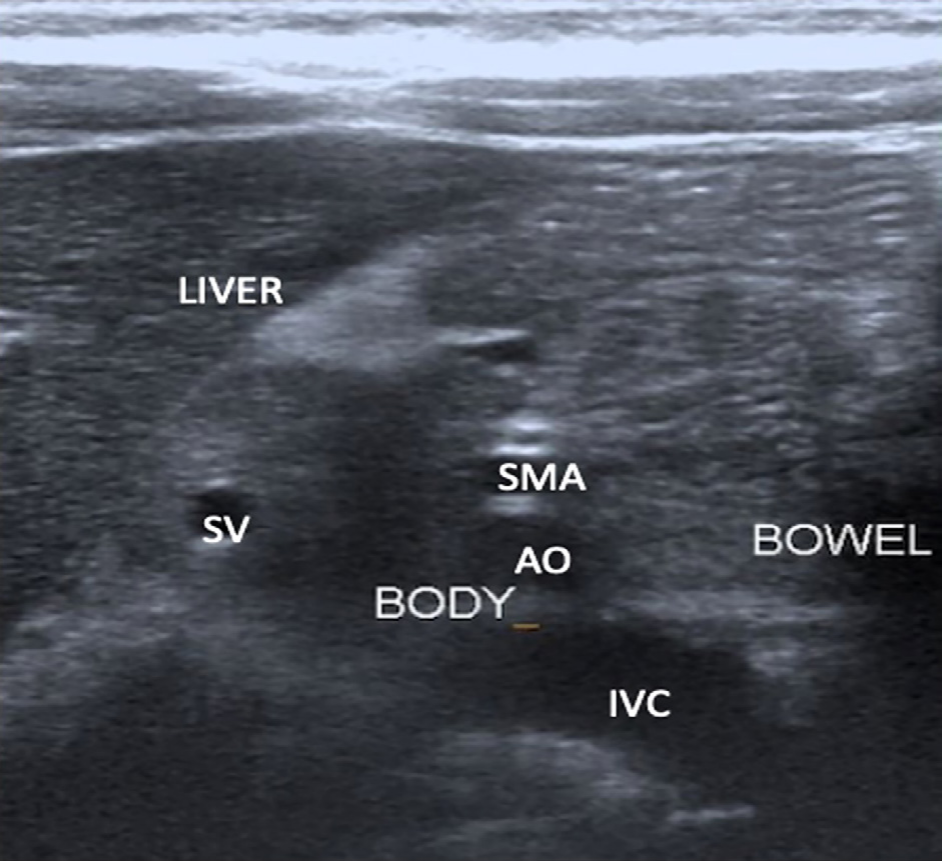
Transabdominal ultrasound midsagittal plane showing normal body and head of pancreas. **SV:** splenic vein, **AO:** abdominal aorta, **SMA:** superior mesenteric artery, **IVC:** inferior vena cava.

Patient was subsequently referred to the paediatric surgical team. An urgent ultrasound abdomen performed to rule out pancreatic or duodenal injury revealed suspicious injury at the pancreatic tail as illustrated in Figure 1 and Figure 2. Other segments of the pancreas were normal without focal collection, pancreatic duct dilatation or peripancreatic free fluid. The duodenum was normal with normal peristalsis. Based on physical signs, elevation of amylase level and concurrent positive ultrasound finding, the diagnosis was revised to traumatic pancreatitis. The patient was admitted to the paediatric surgical ward for observation. Patient was kept nil by mouth with intravenous drip. Pain was managed with intravenous morphine infusion. Intravenous Co-amoxiclav 1.2g was initiated. Due to ongoing pain, she was transferred to the paediatric high dependency unit on day two of hospitalisation. Serial liver enzymes monitoring on day three showed normalisation of ALP level to 129 U/L and improvement of amylase level to 814 U/L. A repeat ultrasound of the abdomen on day six of hospitalisation revealed normal and homogenous head, body and proximal tail of pancreas. However, the distal tail was obscured by bowel gas. Patient completed IV antibiotics and was pain free. She was subsequently discharged home after eleven days of hospitalisation with tablet paracetamol 500mg pro re nata. The patient remained asymptomatic one-month post trauma during her follow-up visit at the paediatric surgical clinic. Repeated ultrasound as outpatient revealed normal pancreas with no peripancreatic fluid or pseudocyst. Consequently, she had further follow-ups at four months interval twice. She then defaulted her follow-up one year later.

## Discussion

Blunt pancreatic injury can prove fatal if not detected, thus a high degree of clinical vigilance is needed since early symptoms are almost always non-specific [1]. Young children are prone to pancreatic injuries more than adults due to their anatomical features of flatter diaphragms, thinner abdominal walls, and higher costal margins. The pathophysiology involves the pancreas being pressed against the vertebral column resulting in compression and contusion of the tissues or the pancreatic duct [1].

This child presented with typical clinical triad of pancreatic trauma which are upper abdominal pain, leucocytosis and elevated serum amylase level [4]. Several studies have looked at the significance of pancreatic enzymes for the early diagnosis and prognosis of pancreatic trauma [5, 6]. Serum amylase level has low sensitivity in pancreatic injury especially during early phase whereby it was found to be non-diagnostic within six hours or less of trauma [6]. Moreover, serum amylase level is not specific to pancreatic trauma as it can be raised in injuries to the salivary gland, head and face, in duodenal and hepatic trauma, and intoxicated patients [4]. A combination of serum amylase and lipase would offer a better option in predicting pancreatic cell damage with 85% sensitivity and 100% specificity [6]. Amylase level does not indicate the severity of pancreatic injury [4]. A multicentre review of nine paediatric trauma centres over five years in the United States revealed that neither initial nor peak amylase level correlated with grade of injury, predicted length of stay or mortality. However, maximal amylase level was highly predictive of pseudocyst formation [5]. 

Predicting pancreatic injury in trauma patients with elevated pancreatic enzymes is hard, thus a scoring system will benefit in identifying high risk patients likely to develop pancreatic injury. A decision-making algorithm using a decision tree was recently proposed in a study limited to adult patients. It achieved an accuracy of 97.9% (sensitivity of 91.4% and specificity of 98.3%) in predicting pancreatic injury using amylase, lipase and glucose level, neutrophils percentage, shock index and presence of abdominal injury as part of the assessment [7]. From the ultrasound scan, the child was suspected to have tail of pancreas injury with normal pancreatic ducts. Pancreatic injury in children can be diagnosed using ultrasound, CT scans, magnetic resonance cholangiopancreatography (MRCP) or endoscopic retrograde cholangiopancreatography (ERCP) [4]. Ultrasound is the first modality in diagnostic imaging for haemodynamically stable patients. It is used to identify other organ injuries such as liver and spleen apart from fluid collection in the pancreas [1]. Pancreatic injuries are difficult to be diagnosed even with technically adequate sonograms [4]. According to Gupta* et al*. CT is the radiographic examination of choice for hemodynamically stable patients with abdominal trauma [8]. It provides the safest and most comprehensive means of diagnosis of a traumatic pancreatic injury. Unlike other abdominal injuries, CT has its limitation in detecting pancreatic injury whereby the pancreas could appear normal in 20 to 40% of patients when it is performed within 12 hours after trauma [4]. This is because, the changes are subtle and usually secondary findings are always relied on to diagnose pancreatic injury. The visualisation of injury will be more difficult with the existence of co-injuries.

A clinical prediction rule comprising of Glasgow coma scale, evidence of abdominal wall trauma or seat belt sign, abdominal tenderness, complaints of abdominal pain, vomiting, thoracic wall trauma, and decreased breath sounds) was able to identify a group of children at very-low-risk (0.1%) for intra-abdominal injury requiring acute intervention. This suggests unnecessary abdominal CT can be averted in very-low-risk group. However, in low risk group as in this child, additional evaluation with serum amylase and ultrasonography is advocated along with consideration of CT [9]. The decision of not doing a CT must be balanced between the possibility of radiation exposure and having a clinically significant pancreatic injury that requires surgical intervention [10]. The child did not undergo a CT, based on her clinical progress and surgeon’s clinical judgement. As a means to avert radiation exposure from CT, contrast-enhanced ultrasonography (CEUS) has been suggested to identify pancreatic injury. The detection rate of CEUS was 95.5% in blunt pancreatic trauma [11]. Nonetheless, CEUS was not available in our setting.

Pancreatic injuries are graded into five categories according to the American Association for the Surgery of Trauma as illustrated in Table 1 [12]. This child most likely has grade I pancreatic injury based on ultrasonography. Almost all grade I injuries are treated conservatively with non-operative management (NOM) [2]. NOM consists of bowel arrest, total parental nutrition and serial imaging with either CT or ultrasound to ascertain injury resolution [13]. NOM failure rate is around 26.0% and injury grades III and above are identified as a predictor of NOM failure [14]. Clinical deterioration and positive findings on repeat sonography must prompt an urgent CT. Pancreatic injury can appear minor initially therefore careful analysis and clinical reassessment are required [10]. Common complications following pancreatic trauma include the formation of pseudocysts and pancreatic fistula [4]. Pancreatic pseudocysts commonly occur after missed injuries to distal pancreas or as a sequela of non-operative management [15]. Our patient had subsequent follow-ups at the paediatric surgical clinic for reassessment of possible complications.

In conclusion, this case report shows that isolated pancreatic injury in children is a rare occurrence and often missed early on as the diagnosis might be challenging. Ultrasound and CT has its limitations in detecting pancreatic injury. A conservative approach can be engaged in patients with low grade injury. Nevertheless, serial assessment with imaging is essential to look for evolution of pancreatic injury and follow-up of non-operative management.

**Table. 1:** American Association for the Surgery of Trauma classification on pancreatic injury

**Grade**	**Injury**	**Description**
**I**	Haematoma	Minor contusion without ductal injury
	Laceration	Superficial laceration without ductal injury
**II**	Haematoma	Major contusion without ductal injury or tissue loss
	Laceration	Major laceration without ductal injury or tissue loss
**III**	Laceration	Distal transection or pancreatic parenchymal injury with ductal injury
**IV**	Laceration	Proximal transection or pancreatic parenchymal injury involving the ampulla
**V**	Laceration	Massive disruption of the pancreatic head

## Conflicts of Interest:

None declared.
